# Experimental Study on the Optimization of the Autoclave Curing Cycle for the Enhancement of the Mechanical Properties of Prepreg Carbon–Epoxy Laminates

**DOI:** 10.3390/polym16010047

**Published:** 2023-12-22

**Authors:** Soňa Rusnáková, Michal Grunt, Milan Žaludek, Jakub Javořík, Barbora Kotlánová

**Affiliations:** Department of Production Engineering, Faculty of Technology, Tomas Bata University in Zlín, Vavrečkova 5669, 76001 Zlín, Czech Republic; zaludek@utb.cz (M.Ž.); javorik@utb.cz (J.J.); b_kotlanova@utb.cz (B.K.)

**Keywords:** autoclave, composite curing, cure cycle, carbon–epoxy prepreg, mechanical properties

## Abstract

In this study, the influence of the technological parameters of autoclave curing on the resulting mechanical properties of laminates was investigated. The main criterion for optimizing the curing was to extend the processing window with a lower prepreg viscosity. At the same time, the issue of setting the pressure level before the heat ramp to the final cure temperature was also addressed. An experimental method of measuring the indentation viscosity of the prepreg was used to determine the viscosity profile. Despite the experimental nature of the method, the reliability of this method for rapid approximate identification of the processing window of the prepreg was verified by the results of the study. Several laminates with the same ply orientation were produced using the selected cure cycles, from which test specimens were cut with a water jet and inspected by confocal microscopy. The mechanical properties of tension and flexure were measured within the individual curing cycles using tests according to ISO standards. The data reported demonstrate that the experimental method of optimizing the curing parameters has successfully increased the selected mechanical properties. The resulting mechanical properties of the laminates were enhanced by up to 20% compared to the non-optimized cure cycle. The influence of the type of cure cycle on the resulting thickness of the cured laminate was evaluated in this study.

## 1. Introduction

In several industrial sectors, such as automotive and aerospace, there is a trend to replace conventional metal parts with composites characterized by high specific strength and stiffness with respect to their weight [1]. The properties of these composite parts largely depend on the type of manufacturing technology and the settings of its parameters [2,3]. Each processing technology has its characteristics and is suitable for a specific type of part [4,5]. The essential predispositions of the technology can be worked with to a certain extent and achieve above-average results by setting the parameters correctly [6]. In industrial practice, efforts are being made to improve and develop constantly. Manufacturers of composite parts are making their technologies lean; reducing energy consumption, processing times, and cost; or increasing product parameters. However, the crucial criteria are producing quality products, meeting customer needs, and being competitive. Autoclave processing remains an indispensable manufacturing technology of one-sidedly visual parts, among others [7]. The technology allows for the setting, controlling, and monitoring of temperature and pressure over time, i.e., all the necessary parameters for successfully curing a laminate [8,9].

The work scope is often associated with criteria such as meeting a certain part thickness, weight, stiffness, strength, and temperature resistance. The industrial development of a composite part consists of many stages. Individual solvers of composite parts rely on their theoretical and practical knowledge. During this time, communication among specialists is essential, as the inputs and outputs of their activities influence each other. Each company has its standardized suppliers and materials with which it works. Standard technological procedures have been set for these materials, which have been developed in the past. Thus, a significant part of technological development has already been completed in the context of bringing new products to market. During the prototype phase, minor quality defects of the product are fine-tuned. Complications arise in the case of a non-standard task when higher quality requirements for parts are requested, whether it is the mechanical properties or the appearance of the part, chemical or temperature resistance, or compliance with geometric dimensions within the required tolerance [10].

This study summarizes beneficial results for both the scientific and industrial spheres. In the application field, it is possible to increase the strength and stiffness of the laminates thanks to this approach. Additionally, increasing the number of layers while maintaining the laminate thickness is possible. This phenomenon is achieved by the effective reduction in the volume ratio of the matrix to the share of reinforcement. During the design phase of a composite part, the load or its deformation is often considered [5]. Then, the material is selected, and its use in the construction of the part is optimized. If, for example, a design requirement arises for using carbon fabric with higher strength or stiffness, an expensive purchase with an extended delivery time can be made, not only suspending development and prototyping, but also increasing costs. Working with a new type of material brings new types of defects that must be dealt with by development engineers. Based on the results of this work, it is possible to increase the selected mechanical properties by several tens of percent when using the same type of prepreg. 

A wide range of prepregs are saturated to a certain percentage of matrix content to reinforcement, and during curing, it is not the goal to change this ratio [11,12]. As a result, it is possible to shorten the curing cycle, as it consists of only one heating, dwell, and cooling. With this method of curing, some prepregs may exhibit the formation of visual (and structural) defects, such as surface or interlaminar porosity and poor layer consolidation, even in cases where prepregs have a low percentage of solvents. Defects can be caused by both insufficient pressure (or its timing) relative to the hydrostatic pressure of the resin and by the fact that insoluble particles can form in epoxy systems at certain temperatures, which then become trapped in the form of vapors often in gaps caused by weaving, or within the layer itself or between them. Furthermore, it can also be a matter of the effect of insufficient layer consolidation. To prevent the occurrence of such defects, it is necessary to optimize the lamination or curing processes [13,14].

In the past, several significant studies have been conducted on the autoclave curing of laminar composites, which are still relevant in current research and industrial applications. This study builds upon previous research by Hernández, Sket, González, and Llorca, who investigated the optimization of heating to minimize the internal porosity of laminates [8]. Additionally, Liu, Zhang, Wang, and Wu studied the setting of pressure during curing to achieve the same goal [13].

In this study, a simple, practical approach is established for characterizing the viscosity of the prepreg during the temperature cycle. The work also deals with the influence of the pressure profile setting during the first isotherm (in the case of a cycle with two isothermal dwells) on the resulting mechanical properties. The work also describes the design advantages of selected pressure profile settings.

## 2. Materials and Methods

### 2.1. Material Selection and Lamination

For the experiments, a prepreg from G. Angeloni GG204P IMP503Z (Venice, Italy) was selected. This prepreg is reinforced with carbon fabric in a plain weave with a nominal areal weight of 220 g/m^2^ [15]. The matrix is an epoxy system with high transparency, making it suitable for visual parts. The epoxy has good mechanical properties and increased UV resistance. Above all, the resin is suitable for processing in a press, autoclave, or oven with the use of a vacuum and can be cured in a broader range of temperatures. After curing, laminates from this prepreg have a glass transition temperature (T_g_) of 120 °C. Other properties of the material are listed in Table 1 [16].

Laminates for the experiments were architected as cross-ply laminates with a stacking sequence of [0/90/0/90/$\overset{-}{0}$]s. This composition was chosen concerning the character of the mechanical tests and the estimated final thickness of the laminate.

**Figure 1:**
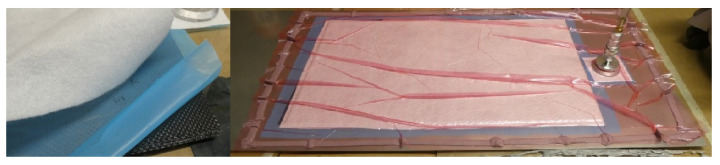
Lamination process (**left**: the stack of the prepreg covered with perforated release film and breather cloth; **right**: debulking of the laminate prior to curing).

Prior to processing the prepreg, the selected glass mold was cleaned mechanically, with a scraper, and chemically using Chem Trend Lusin Clean L 21 (Sibiu, Romania). The mold surface was then treated with a micropore filler Zyvax Sealer GP (Sibiu, Romania) and then separated with Chem Trend Chemlease 2191 W (Sibiu, Romania). During lamination, 15 min debulks were carried out every three layers to ensure the necessary consolidation of the layers to remove any air between the layers. Before curing, laminates were covered with perforated release film, a breather cloth, and wrapped in a vacuum bag (Figure 1) [18,19].

### 2.2. Characterization of Curing Cycle Optimization

This study aims to optimize the cycle towards higher mechanical properties and lower amounts of internal pores in the laminate, which are often potential sites for cohesion failures or other defects [20]. The prepreg manufacturer states that the curing cycle specified by them is only a proposal for processing the prepreg and is not the only way to process the raw material properly.

To meet the optimization criterion, it was necessary to extend the processing area when the resin has a low viscosity. At this point, it better impregnates the fibers, allows voids to escape from the laminate, and, in addition, its excess drains into the breather cloth [21]. Here, the course of viscosity measured by the prepreg manufacturer and the experiment [7] was used.

In the study [7], a two-stage heating proved to be effective in minimizing internal pores and thus enhancing the mechanical properties of the laminate.

The design of the pressure profile needed to be clarified. The experimental studies and literature [2,4,7,8,13,18,21] differ in interpreting the pressure profile setting, respectively, its setting from the beginning of the cycle to the end of the first isotherm. There is space to determine which pressure profile is the most suitable for optimal properties. The following curing cycles differ in the pressure setting from the beginning of the cycle (first heating ramp and first isothermal dwell) to the dwell at the curing temperature, when the pressure value is always the maximum possible (6 bar). This value is set until the temperature in the autoclave drops to approximately 85 °C, then it starts to decrease smoothly to 0 bar.

The vacuum setting was modified so the vacuum pump would not work unnecessarily. The theoretical study shows that a vacuum is essential to use in order to remove internal volatiles. However, volatiles cannot be removed if the viscosity of the resin increases (begins to form a spatial network). If it is clear that curing has already begun, there is no point in the product being subjected to vacuum. Thus, the vacuum was deactivated approximately 10 min after reaching the curing temperature.

The proposed course of the temperature cycle was verified and compared using an experimental indentation test method of the prepreg viscosity. The curing cycles were carried out in an autoclave OP Panini S.R.L., model G00300572 [22] (Maranello, Italy).

### 2.3. Viscosity Profile Identification

Within this article, an experimental practical test method for measuring the viscosity profile is published.

**Figure 2:**
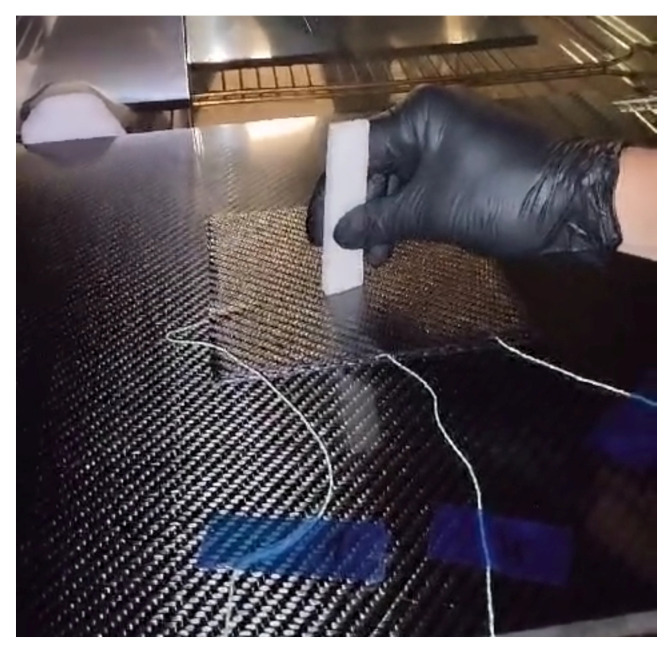
Demonstration of the indentation test of prepreg viscosity.

The method can be called an indentation test of prepreg viscosity; it allows for the monitoring of the curing process of a prepreg laminate by inserting and slightly vibrating an indenting body into the laminate while it is subjected to heating following a temperature profile of the selected cure cycle; see Figure 2. The indenting body can be made of wood, temperature-resistant polymer foam, or cork.

The primary purpose of the test is to examine the viscosity profile of a prepreg and thus define the proper process setting of a curing cycle. The method can be used to determine the gel time of any prepreg with its curing cycle (different heating rates or multiple levels of dwell), from which pressure settings, mold closing time, or individual prepreg batches can be compared.

The test is typically carried out in a conventional oven or on a temperature-controlled press plate. The tested laminate specimen should be subjected to a heating transfer similar to that intended to be used within the final processing method. In the case of thicker future parts or highly reactive systems, it is convenient to perform the test with a laminate of the appropriate thickness, as this will ensure that the test captures the effects of exothermic phenomena. A scale and unit for this test has been created. The unit of the test in this study is called the index of indentation viscosity of a prepreg, marked as *i*, and takes values from 1 to 0.

The index of indention viscosity (*i*) is a dimensionless quantity used to quantify the state of a prepreg and the ease with which it can be deformed under load (Table 2). The higher the index, the more viscous the prepreg is, and the more difficult it is to deform. A low index indicates that the prepreg is less viscous and is easier to deform. The test result can be interpreted as a plot of the indention viscosity index of the prepreg over time.

Although this test may involve subjective evaluations, with expertise, it is possible to obtain repeatable and reliable results.

### 2.4. Testing of Mechanical Properties

To evaluate the influence of the setting of the technological parameters of curing cycles on mechanical properties, tensile tests ISO 527-4 and flexural tests ISO 14 125 were selected [23]. The evaluation is focused on the maximal strength and modulus of elasticity of the laminates.

#### 2.4.1. Three-Point Flexural Test

**Figure 3:**
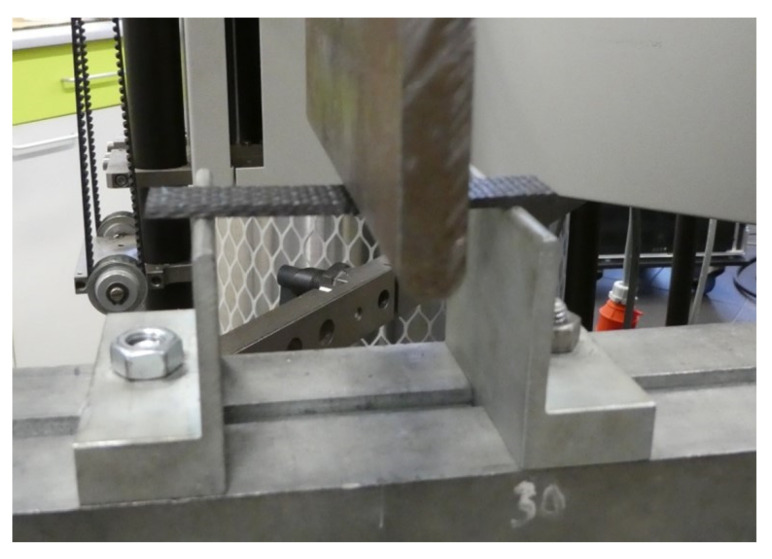
Three-point bending test setup.

Test specimens of dimensions 80 × 15 × 2 mm were cut from each cured plate using a water jet to determine flexural properties. The following is a comparison of the individual curing cycles in terms of the evaluated flexural properties. The test was carried out according to the ISO 14 125 standard for three-point bending on a Zwick 1456 testing machine (Figure 3) [24].

First, the test specimen is placed symmetrically relative to the bending pin on supports spaced 64 mm apart. Then, the pin bends the specimen until it breaks. During this, the material properties in bending are measured based on the following equations:

(1) $$\mathrm{Flexural}\;\mathrm{stress}\;(\sigma_{f})=\frac{3\cdot F\cdot L}{2\cdot w\cdot t^{2}}$$

(2) $$\mathrm{Flexural}\;\mathrm{modulus}\;(E_{f})=\frac{L^{3}}{4\cdot w\cdot t^{2}}\cdot \left(\frac{∆F}{∆y}\right)$$

where F is the load (N), L is the support span (mm), w is the width of the specimen (mm), t is the thickness of the specimen (mm), ΔF is the difference between two forces at two different deflections (N), and Δy is difference between two deflections at two different forces (mm).

#### 2.4.2. Tensile Test

**Figure 4:**
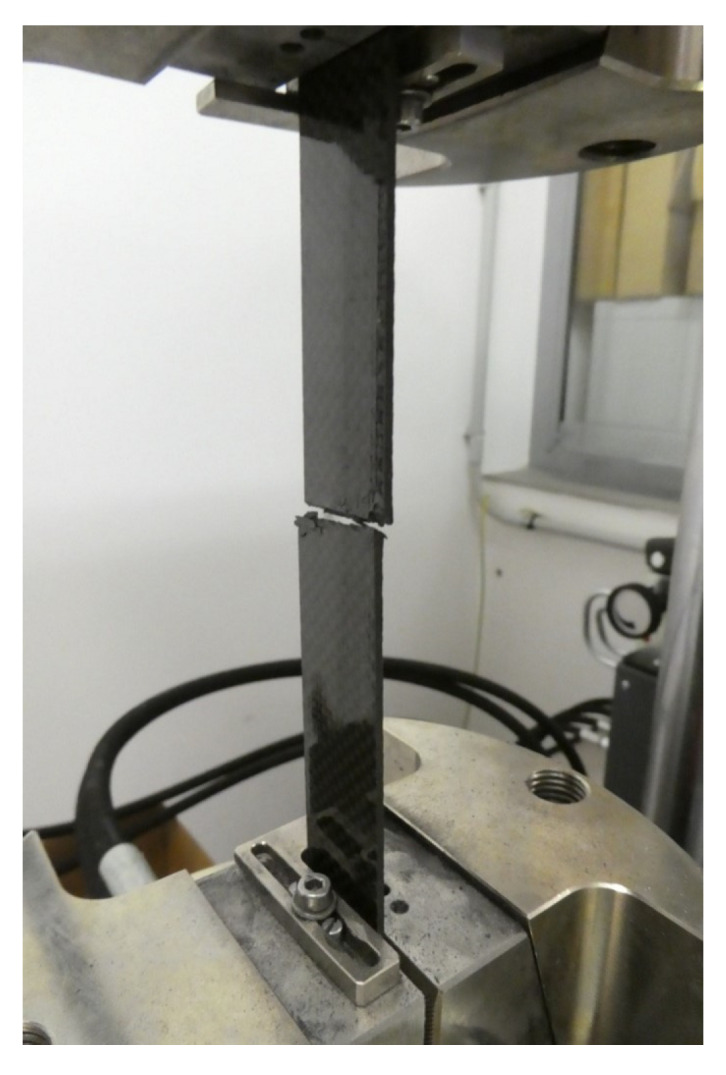
Specimen after the tensile test.

From each cured laminate, 5 test specimens of dimensions 250 × 20 × 2 mm, intended for tensile testing, were cut using a water jet. The test was carried out according to the ISO 527-4 standard on a Zwick Vibrophore 100 testing machine. Hydraulic jaws secured the test specimen with a pressure of 30 bar. At the beginning of the test, an extensometer was attached to the specimen, which measured the tensile modulus in the range of 0–0.25% L_0_. After that, it was removed from the specimen, and the test continued without it. The tensile properties were measured until the specimens broke (Figure 4) [25].

Tensile stress and modulus were calculated by the following equations:

(3) $$\mathrm{Tensile}\;\mathrm{stress}\;\left(\sigma_{t}\right)=\frac{F_{t}}{w\cdot t}$$

(4) $$\mathrm{Tensile}\;\mathrm{modulus}\;(E_{t})=\frac{\sigma_{t}}{\varepsilon }=\frac{F_{t}\cdot L_{0}}{w\cdot t\cdot (L-L_{0})}$$

where F_t_ is the load (N), w is the width of the specimen (mm), t is the thickness of the specimen (mm), ε is the strain of the specimen (%), L_0_ is the initial gauge length (mm), and L is the final length (mm).

## 3. Results and Discussion

### 3.1. Cure Cycle Optimization

The indentation test method was chosen to optimize the temperature profile of the manufacturer’s proposed curing cycle. Initially, the index of indentation viscosity for the curing cycle proposed by the prepreg manufacturer was examined.

The cure cycle suggested by the manufacturer (MStd) consists of a ramp with a heating rate of 2.1 °C from 20 °C to 125 °C, continues with a dwell at 125 °C for 60 min, and is followed by a cooling phase with a rate of 1.6 °C/min.

**Figure 5:**
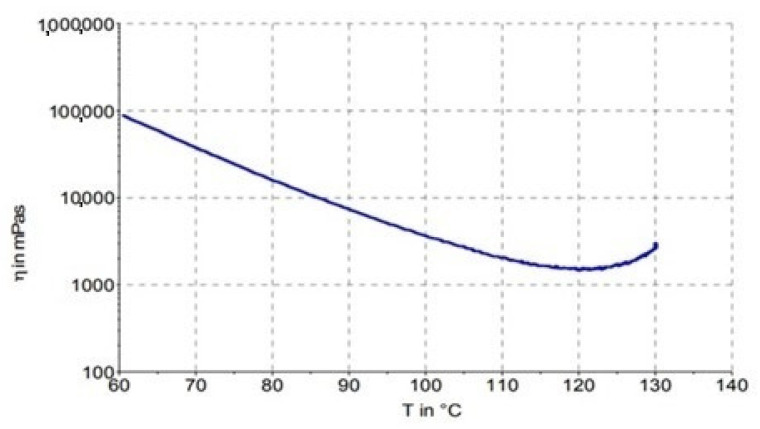
Viscosity vs. temperature behavior of IMP503Z resin measured by the manufacturer of the prepreg.

The course of viscosity, depending on temperature (Figure 5), shows at what temperature the viscosity is sufficiently low and at the same time, it is not close to the point where it starts to cross-link. The strategy was to prolong a region of lower viscosity so the laminates have more time to become consolidated and for the vapors to be evaporated. The optimized cure cycle strategy was established by the viscosity course depicted in the prepreg material’s technical datasheet (Figure 5). The viscosity of the IMP503Z resin was measured by a begin-cone-plate rheometer. Measurement began at the temperature of 60 °C. It was conducted with a frequency of 0.2 Hz and a heating rate 3 °C/min.

In order to optimize the cure cycle and thus the selected laminates’ mechanical properties, the processing window should be prolonged [26]. The assumption was to split the cure cycles into two heat ramps, two dwells, and one cooling phase. The first heat ramp of optimized cure cycles was performed with a heating rate of 2.5 °C/min to 100 °C from 20 °C, followed by a 20 min dwell, and then the second ramp with a heating rate of 5 °C/min to the final dwell temperature of 125 °C. The second dwell had, in all cases, a duration of 60 min. Subsequently, the cooling phase commenced with a cooling rate of 1.6 °C/min.

**Figure 6:**
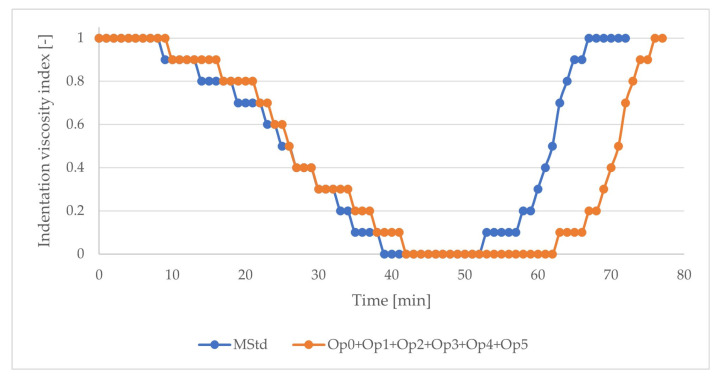
The course of the index of indentation viscosity of the prepreg (blue: MStd; orange: Op0, Op1, Op2, Op3, Op4, Op5).

The result of the test demonstrates the positive effect of the inclusion of two-stage heating on the extension of the processing window (Figure 6) without extending the curing cycle (the total curing cycle time remained 165 min). In this case, it was possible to extend the plateau area of the lowest indentation viscosity index by 60%. The proposed optimization of the temperature cycle was incorporated into all optimized curing cycles (Op0, Op1, Op2, Op3, Op4, and Op5).

**Figure 7:**
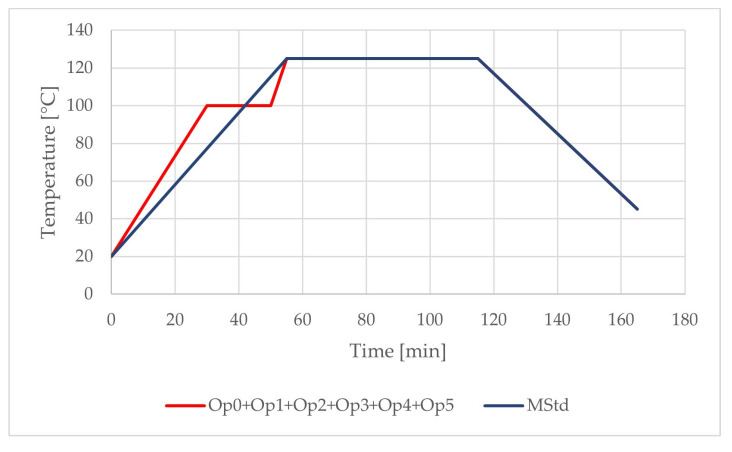
Comparison of the temperature profiles of the curing cycles (blue: MStd; red: Op0, Op1, Op2, Op3, Op4, and Op5).

The realized temperature profiles of the curing cycles can be compared in Figure 7 and Table 3. Cure cycle MStd (manufacturer´s standard cure cycle) consists of the direct ramp-up to the final dwell temperature and cooling stage. All other optimized curing cycles consist of the same temperature profiles, which include two-stage heating ramps, two dwells, and one cooling phase. Thus, the cure cycles differ only before reaching the final dwell temperature.

**Figure 8:**
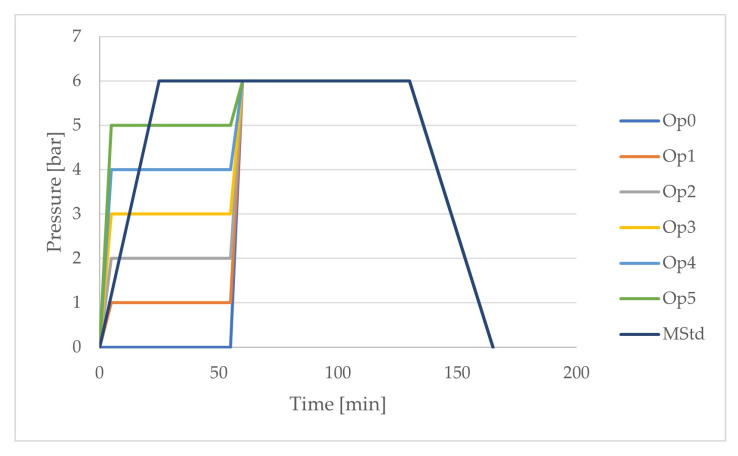
Comparison of the pressure profiles of the curing cycles (dark blue: MStd, light blue: Op0, orange: Op1, grey: Op2, yellow: Op3, blue: Op4, green: Op5).

Based on the theoretical and experimental study, curing cycles were designed to determine the optimal setting of curing parameters with respect to the selected mechanical properties of the laminate. Cycles with the names Op0 to Op5 and MStd were compared. The letters in the names of the curing cycles with the initial letter O express the value of the pressure in bars during the first heating and the first dwell of the optimized temperature course of the curing cycle. The curing cycle with the name MStd contains the curing parameters recommended by the material manufacturer. The pressure courses are shown in Figure 8 and Table 4.

The maximum pressure of 6 bars was set for all cure cycles as the final temperature dwell commenced. The individual curing cycle optimizations differ in the pressure setting during the first ramp and the first dwell.

All curing cycles also included vacuuming, which took place from the beginning to the first third of the dwell at the highest temperature, after which the vacuum was deactivated. The vacuum was not applied due to the assumption that the system was already cross-linking and for energy consumption reasons [27].

### 3.2. Visual Inspection of Cured Laminates

**Figure 9:**
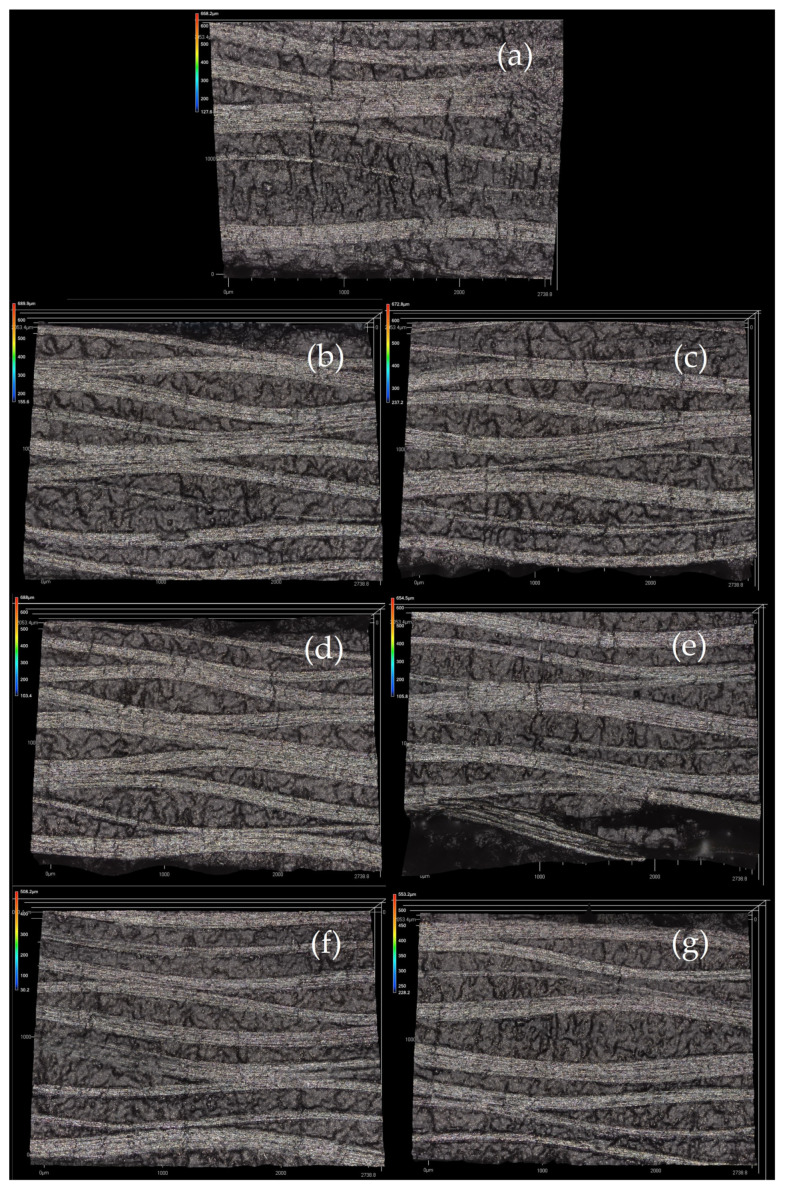
Confocal microscopy scans of the cured laminates at 5× magnitude ((**a**) MStd, (**b**) Op0, (**c**) Op1, (**d**) Op2, (**e**) Op3, (**f**) Op4, (**g**) Op5).

In order to gain a closer understanding of the internal structure of the fabricated laminates, observations were made using a Keyence VK1000 confocal microscope (Mechelen, Belgium). Locations around the perimeter of the water-jet-cut test specimens were observed randomly. Scans were made at 5× magnification (Figure 9). Prior to the scanning, sections of the laminates were inspected visually for any flaws. However, no suspected defect was indicated on the specimens, so their surfaces were observed randomly with the microscope.

The images obtained at 5× magnification reveal the stacked and waved layers resulting from the plain weave and technology parameters. It is known that by curing in an autoclave, the individual plies of a laminate can be very well consolidated by pressure, and thus the crimping can be minimized. The degree of fiber crimp limits the mechanical properties. Additionally, no flaws like voids, porous surfaces, resin-rich areas, or kinked fiber tows were found on the specimens, which supports the claim that all cure cycles were well established.

### 3.3. Effect of Cure Cycles on Mechanical Properties

To evaluate the influence of the setting of the technological parameters of curing cycles on mechanical properties, tensile tests in accordance with ISO 527-4 and flexural tests in accordance with ISO 14 125 were selected. The evaluation is focused on the strength and modulus of elasticity of the given load.

The Zwick 1456 universal tensile testing machine was used to carry out the three-point flexure tests. The accuracy of the force measurement was within ±1.0% within a range of 20 kN, and the accuracy of the distance measurement of the test jaws was 0.09 μm.

The material’s tensile properties were measured using a Zwick Vibrophone 100 universal tensile machine(ZwickRoell s.r.o., Brno, Czech Republic). The machine has a force measurement accuracy of ±1.0% within a range of 100 kN and a test jaw distance measurement accuracy of 0.09 μm.

Both test machines have a force resolution of 0.1 N and were calibrated according to ISO 7500-1 at the time of measurement.

#### 3.3.1. Evaluation of Flexural Tests

The statistical evaluation of the measured data from the three-point flexural test is shown in Table 5 and Table 6. Evaluated were the following: $\overset{-}{x}$ is the arithmetic mean, $s_{\overset{-}{x}}$ is the standard deviation of the data set, u_a_ is the type A evaluation of uncertainty, and $v_{\overset{-}{x}}$ is the coefficient of variation.

**Figure 10:**
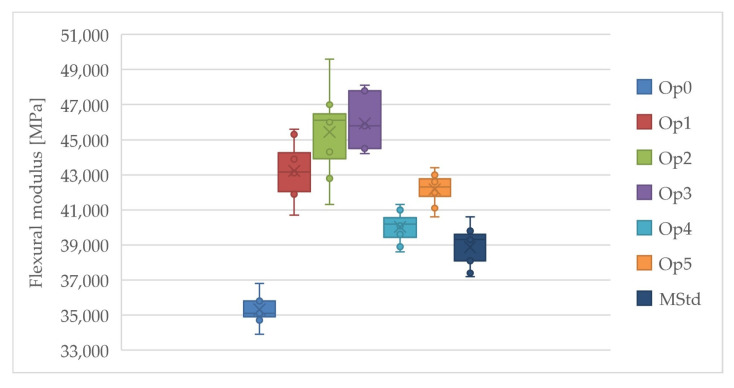
Comparison of the flexural modulus of the cured laminates (dark blue: MStd, blue: Op0, red: Op1, green: Op2, purple: Op3, light blue: Op4, orange: Op5).

Curing cycles Op3 and Op2 achieved the highest values of the average flexural modulus, while the lowest values were achieved by Op0, MStd, and Op1 (Figure 10). Very high values of the average flexural modulus were also achieved by cycle Op1. The average flexural modulus values of curing cycles Op4 and Op5 are statistically located roughly in the middle of the range of all measured values. These cycles have the lowest standard deviation. The lowest uncertainty of type A measurements was found for curing cycles Op0, Op4, and Op5. Conversely, MStd, Op3, Op1, and Op2 exhibited higher values in that order. The low coefficient of variation values demonstrate the minimal dispersion in the measured values and the high accuracy of the calculated arithmetic mean.

The results of the evaluation demonstrate adequate property consistency throughout all curing cycles. The study reveals that pressure significantly impacts increasing the flexural modulus to a maximum limit of 3 bar. Below 1 bar, pressure values result in an abrupt modulus decrease, while on the other hand, values above 3 bar manifest a modulus decrease below the levels obtained at 1 bar pressure.

**Figure 11:**
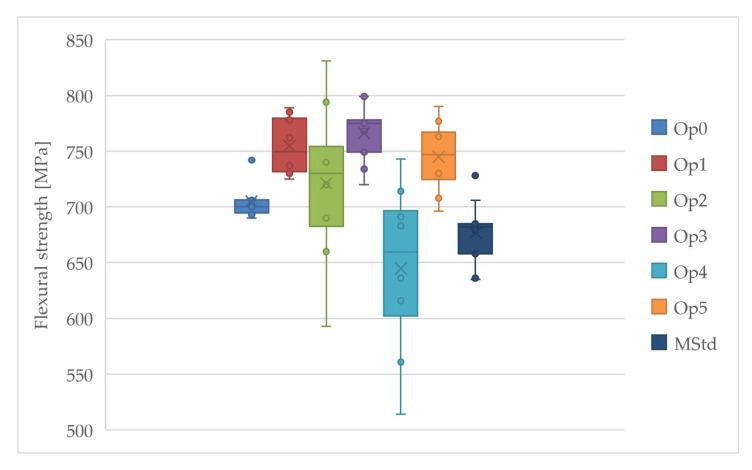
Comparison of the flexural strength of the cured laminates (dark blue: MStd, blue: Op0, red: Op1, green: Op2, purple: Op3, light blue: Op4, orange: Op5).

The highest value of the average flexural strength was also achieved using curing cycle Op3, while the lowest value was achieved by curing cycles Op4 and MStd (Figure 11). Very high values are also reached by the average flexural strength at Op1 and Op5. The curing cycle Op2 contains the most extensive dispersion of the measured values. The average flexural strength values were achieved by curing cycle Op0 within the set of all measurements, but the range of the values of this cycle is the lowest.

Low levels of the uncertainty of type A measurements were detected for all curing cycles. However, the Op2 and Op4 curing cycles exhibited slightly higher values, each presenting a coefficient of variation that approaches 10%. Such a result suggests that the accuracy of the testing may have deviated when determining the mean flexural strength value. Furthermore, Figure 11 shows a significant dispersion of values. Lower dispersion values were observed for the remaining cycles.

The modification of technological parameters from the manufacturer’s specifications had a positive effect on the flexural strength in all curing cycles, except for Op4.

**Figure 12:**
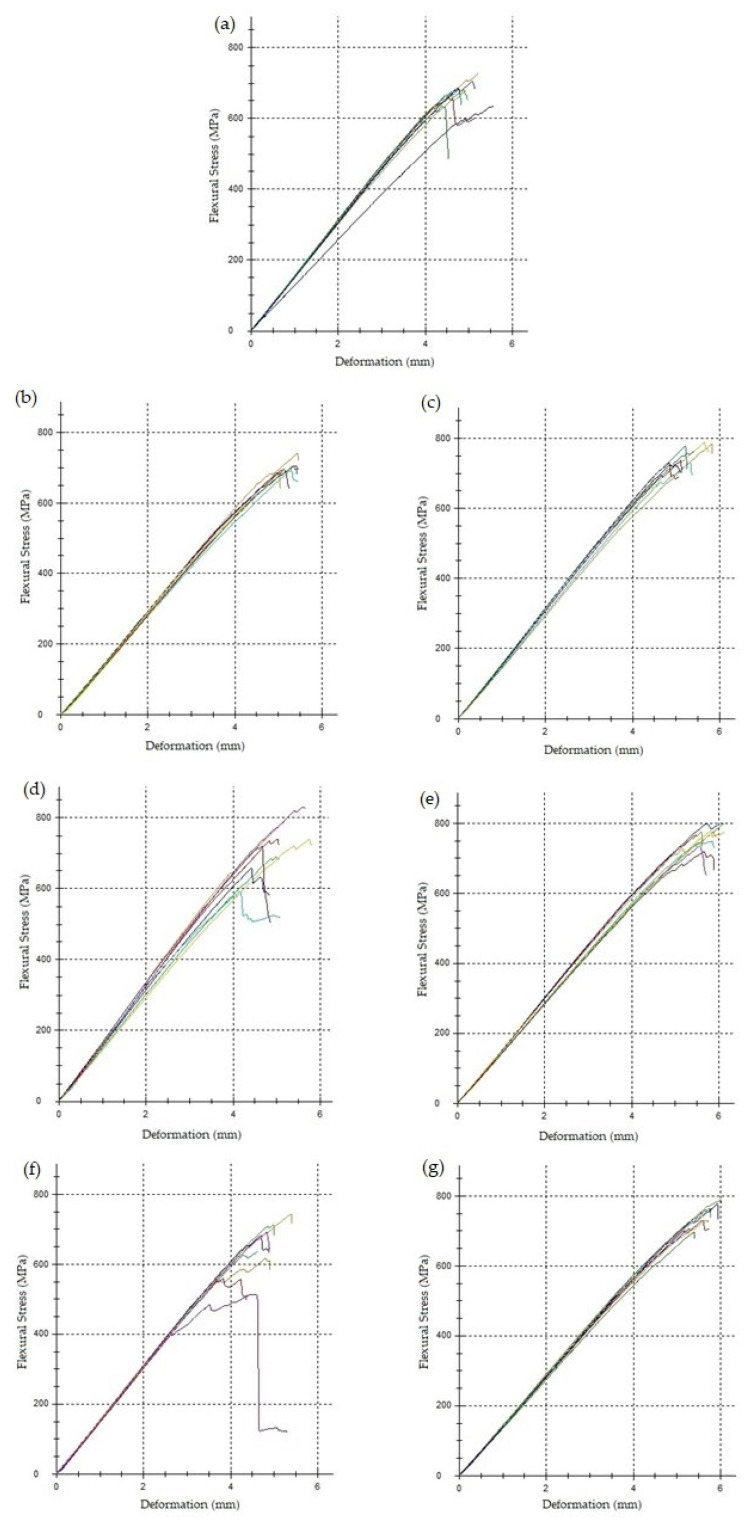
Flexural stress–deformation curves ((**a**) MStd, (**b**) Op0, (**c**) Op1, (**d**) Op2, (**e**) Op3, (**f**) Op4, (**g**) Op5).

All manufactured laminates exhibited standard linear behavior under the load, as shown in Figure 12.

#### 3.3.2. Evaluation of Tensile Tests

The statistical evaluation of the measured data from the tensile test is shown in Table 7 and Table 8. Evaluated were the following: $\overset{-}{x}$ is the arithmetic mean, $s_{\overset{-}{x}}$ is the standard deviation of the data set, u_a_ is the type A evaluation of uncertainty, and $v_{\overset{-}{x}}$ is the coefficient of variation.

**Figure 13:**
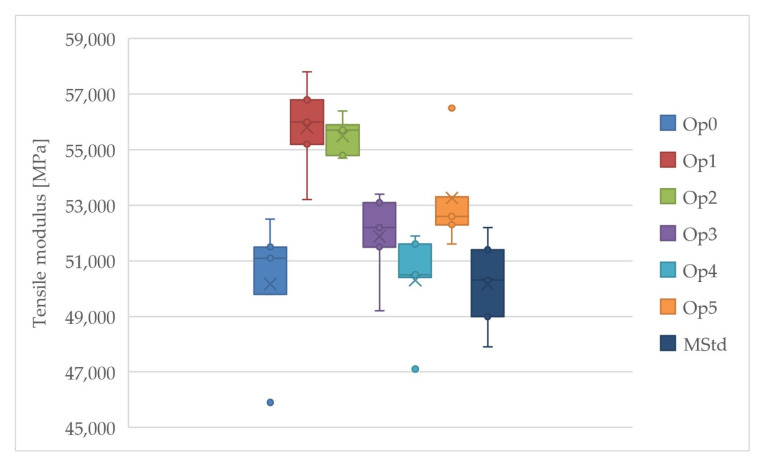
Comparison of the tensile modulus of the cured laminates (dark blue: MStd, blue: Op0, red: Op1, green: Op2, purple: Op3, light blue: Op4, orange: Op5).

Curing cycles Op1 and Op2 achieved the highest values of the average tensile modulus, while the lowest values were achieved by Op0, MStd, and Op4 (Figure 13). Cycle Op2 also contains the smallest estimate of the sample standard deviation of the tensile modulus. Very high values of the average tensile modulus were also achieved by cycles Op3 and Op5. These curing cycles are located approximately in the middle of all measured values.

The Op2 curing cycle exhibits the lowest standard deviation, type A measurement uncertainty, and coefficient of variation. The dispersion and variation characteristics are slightly more significant for the other cycles performed, but not dramatically. Thus, the results support a significant consistency of the tensile module across all manufacturing strategies.

A significant improvement in tensile modulus, particularly for the Op1 and Op2 optimizations, can be noted. However, the remaining curing cycles show a less substantial increase. In addition, the selected process parameters successfully decreased the tensile modulus dispersion compared to the MStd cycle.

**Figure 14:**
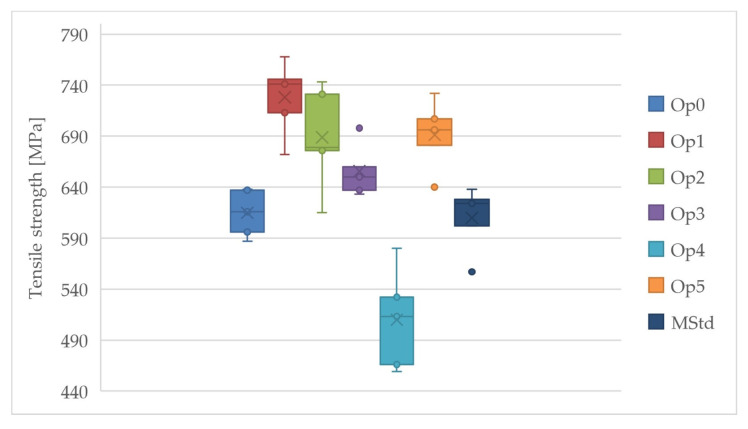
Comparison of the tensile strength of the cured laminates (dark blue: MStd, blue: Op0, red: Op1, green: Op2, purple: Op3, light blue: Op4, orange: Op5).

A comparison of the tensile strength among curing cycles is depicted in Figure 14. The highest value of the average tensile strength was achieved using curing cycle Op1, while the lowest value was achieved by curing cycle Op4. Very high values were also reached by the average tensile strength at Op5, Op2, and Op3. Very similar values of the average tensile strength were measured for curing cycles Op0 and MStd.

Cycles Op2 and Op4 exhibit the highest values of sample standard deviations, type A measurement uncertainty, and coefficient of variation. The higher dispersion of values can also be observed in Figure 14. In contrast, the remaining cycles exhibit comparable sampling standard deviation, coefficient of variation, and type A measurement uncertainty. All realized cycles have a relatively reasonably large dispersion of the measured values, indicating the production’s reliability and the reliability of the measurement itself. Regarding tensile strength, the Op4 cycle exhibits a lower value than the MStd baseline cycle.

**Figure 15:**
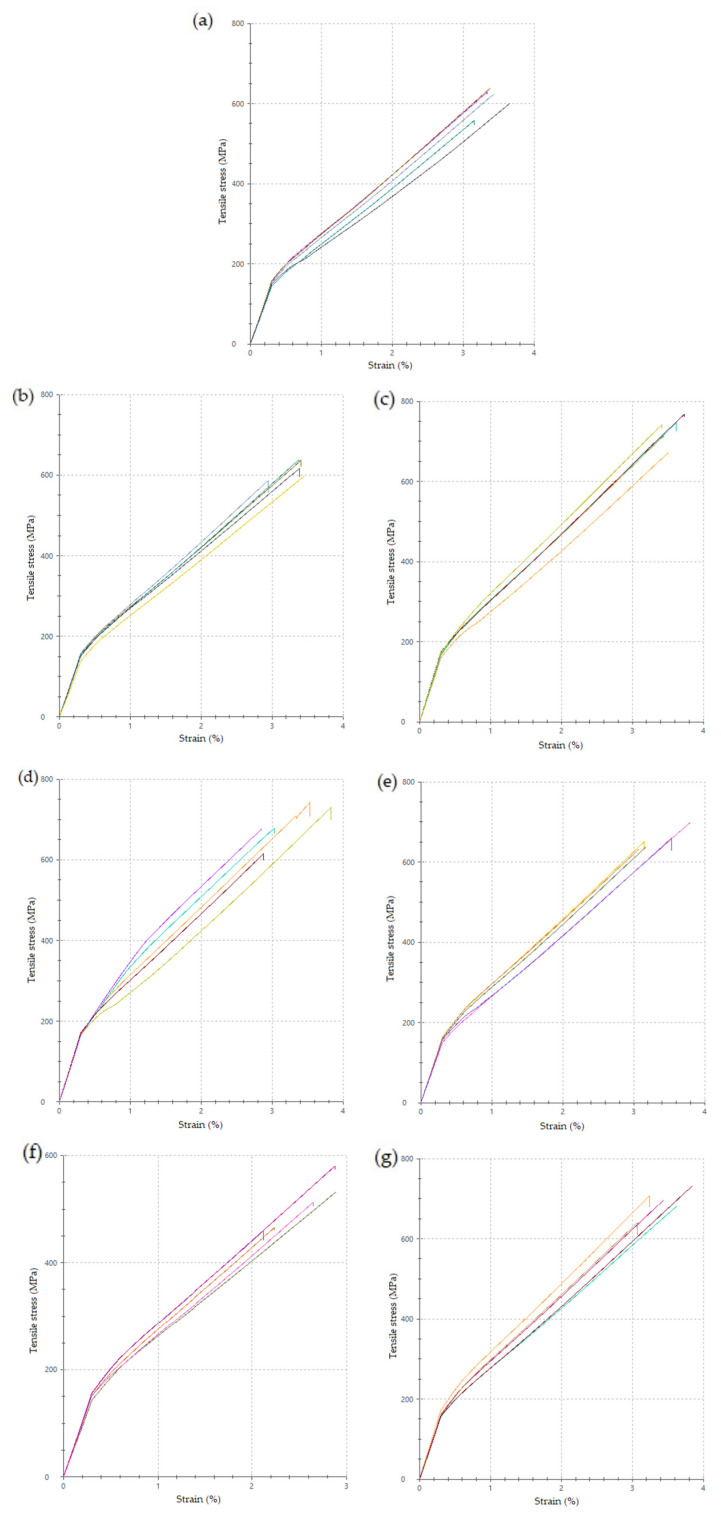
Tensile stress–strain curves ((**a**) MStd, (**b**) Op0, (**c**) Op1, (**d**) Op2, (**e**) Op3, (**f**) Op4, (**g**) Op5).

All manufactured laminates exhibited standard linear behavior under the load, as shown in Figure 15. The change in the slope of the curves is caused by the measurement setup. In the first 0.3% of tensile strain, the slope is measured by the attached extensometer to the tested specimen’s surface; when the strain value is reached, the extensometer is removed, and the measurement continues only with extensometers included in the machine jaws.

### 3.4. Effect of Cure Cycles on Laminates’ Thickness

One of the critical parameters that needs to be considered in the design of a composite part is the final thickness of the cured ply (CPT). It can be calculated by the following formula:

(5) $$\mathrm{Cured}\;\mathrm{ply}\;\mathrm{thickness}\;(\mathrm{CPT})=\frac{t}{n}$$

where t is the thickness (mm), and n is the number of layers (-).

The thickness of the cured laminates varies significantly between the different curing cycles (Table 9). There is a difference of almost 13% in the laminate thickness between curing cycles Op0 and Op2.

**Figure 16:**
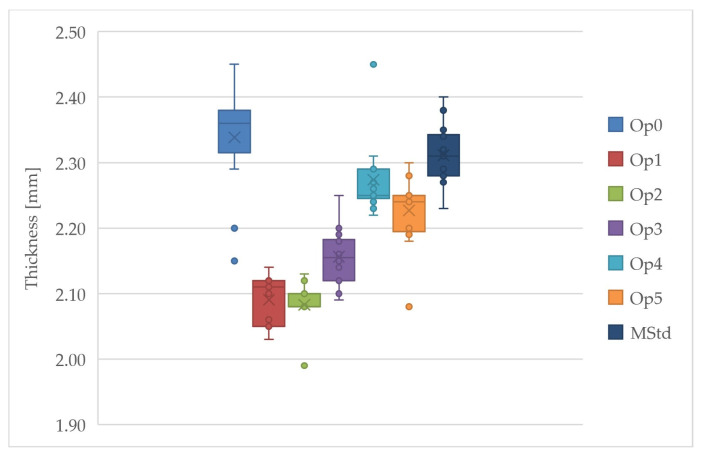
Comparison of the thickness of the cured laminates (dark blue: MStd, blue: Op0, red: Op1, green: Op2, purple: Op3, light blue: Op4, orange: Op5).

A graphical representation of the variation in thickness across the curing cycles is shown in Figure 16. Thicknesses of the specimens enter into all mechanical property evaluations and affect the results substantially. This phenomenon supports the fact that the fiber over matrix volume and mass fraction varies among performed cure cycles.

## 4. Conclusions

The investigation of the pressure profile setting during the autoclave curing of laminates so far indicates that it is advisable to apply pressure and split the heating into two stages. The two-stage heating can substantially extend the processing area of the prepreg. By extending the processing area, it is possible to wet the fibers effectively, consolidate the layers, and extract any volatile fractions. Measurements of the mechanical properties and the resulting laminate thickness show a specific correlation between these quantities. The highest values of mechanical properties were measured at the smallest thicknesses, which resulted in an increase in the percentage of carbon reinforcement relative to the epoxy matrix.

Laminates with carbon reinforcement and epoxy matrix were produced using autoclave processing. The experiment was focused on monitoring the influence of process parameters on the resulting mechanical properties of the laminates, aiming to optimize the mechanical properties of the laminate. The cycle recommended by the prepreg manufacturer (MStd) was compared with experimentally optimized cycles Op0 to Op5.

The experimental method was used to measure the indentation viscosity of the prepreg and optimize the temperature cycle. This approach provided an alternative way to evaluate and compare the processing window with low viscosity. The method also helped determine the optimal pressure application time in accordance with the theory of void formation. The optimal pressure value was determined experimentally, and the resulting laminates were subjected to mechanical testing.

The study’s results show the influence of the setting of process parameters on stiffness and strength in tensile and flexural loading. The specimens produced using cycle Op3 had the highest mechanical properties in flexural loading, and in tensile loading, the specimens produced using cycle Op1. Average flexural strength was improved by 13% and average modulus by 18%, along with the average tensile strength increasing by 19% and the average modulus by 11%, compared to values obtained by a non-optimized cure cycle.

Laminates manufactured through different cycles have different thicknesses. The thinnest laminates were produced through the curing cycles Op1, Op2, and Op3, and the thickest through the cycles Op0 and MStd. Measured average thicknesses of cured laminates vary by up to 12%. By choosing the right curing cycle, it is possible to prosper not only in the field of composite part design, but in other industrial applications. 

This experimental methodology can be effectively applied in the industrial sphere in the structural design of composite parts and in the setting of processing parameters for curing in an autoclave. This study outlines a practical approach to enhancing the mechanical properties of laminates simply by optimizing cure cycle parameters.

## Figures and Tables

**Table 1:** Reference prepreg material properties [17].

Properties of Resin IMP503Z		
Property	Value	Unit
Density	1.15 ÷ 1.25	g/cm^3^
Gel time 125 °C	6 ÷ 9	min
Gel time 110 °C	20 ÷ 26	min
Tack level	intermediate ÷ high	-
Void content	<1	%
T_g_—cured resin	120	°C
Out-life 23 °C	5	weeks
Shelf life −18 °C	1	year
Reference properties of prepreg GG204P IMP503Z cured by compression molding		
Property	Value	Unit
Flexural strength (ASTM D790)	810	MPa
Flexural modulus (ASTM D790)	53,000	MPa
Fiber volume fraction	59	%

**Table 2:** The scale of the index of indentation viscosity of a prepreg with expected values of viscosity.

Expected State of Prepreg Material	*i* (-)	*η* (mPas)
rigid	1.0	N/A
less rigid with high tackiness	0.9	N/A
soft	0.8	N/A
soft to very high viscosity transition zone	0.7	N/A
very high viscosity	0.6	(500,000, 1,000,000)
high viscosity	0.5	(200,000, 500,000)
very high and medium viscosity transition zone	0.4	(100,000, 200,000)
medium viscosity	0.3	(50,000, 100,000)
medium and low viscosity transition zone	0.2	(10,000, 50,000)
low viscosity	0.1	(2000, 10,000)
very low viscosity	0.0	(0, 2000)

**Table 3:** Temperature settings of the performed cure cycles.

MStd		Op0, Op1, Op2, Op3, Op4, Op5	
Time [min]	Temperature (°C)	Time [min]	Temperature (°C)
0	20	0	20
55	125	30	100
115	125	50	100
165	45	55	125
		115	125
		165	45

**Table 4:** Pressure settings of the performed cure cycles.

MStd			Op0	Op1	Op2	Op3	Op4	Op5
Time [min]	Pressure [bar]	Time [min]	Pressure [bar]	Pressure [bar]	Pressure [bar]	Pressure [bar]	Pressure [bar]	Pressure [bar]
0	0	0	0	0	0	0	0	0
25	6	5	0	1	2	3	4	5
130	6	55	0	1	2	3	4	5
165	0	60	6	6	6	6	6	6
		130	6	6	6	6	6	6
		165	0	0	0	0	0	0

**Table 5:** Evaluation of the flexural modulus of the cured laminates.

Flexural Modulus (MPa)							
	Op0	Op1	Op2	Op3	Op4	Op5	MStd
$\overset{-}{x}$	35,350	43,225	45,438	45,922	40,025	42,175	38,867
$s_{\overset{-}{x}}$	864	1682	2585	1618	947	951	1152
u_a_	305	595	914	539	335	317	384
$v_{\overset{-}{x}}$(%)	2.44	3.89	5.69	3.52	2.37	2.26	2.96

**Table 6:** Evaluation of the flexural strength of the cured laminates.

Flexural Strength (MPa)							
	Op0	Op1	Op2	Op3	Op4	Op5	MStd
$\overset{-}{x}$	703	755	721	766	645	745	677
$s_{\overset{-}{x}}$	17	27	75	27	78	34	30
u_a_	6	9	26	9	28	11	10
$v_{\overset{-}{x}}$(%)	2.48	3.55	10.38	3.55	12.17	4.52	4.49

**Table 7:** Evaluation of the tensile modulus of the cured laminates.

Comparison of Tensile Modulus (MPa)							
	Op0	Op1	Op2	Op3	Op4	Op5	MStd
$\overset{-}{x}$	50,160	55,800	55,500	51,880	50,300	53,260	50,160
$s_{\overset{-}{x}}$	2571	1744	731	1675	1907	1911	1744
u_a_	909	616	259	558	674	637	581
$v_{\overset{-}{x}}$(%)	5.12	3.12	1.32	3.23	3.79	3.59	3.48

**Table 8:** Evaluation of the tensile strength of the cured laminates.

Comparison of Tensile Strength (MPa)							
	Op0	Op1	Op2	Op3	Op4	Op5	MStd
$\overset{-}{x}$	615	728	689	656	510	691	610
$s_{\overset{-}{x}}$	23	37	51	26	50	34	32
u_a_	8	13	18	9	18	11	11
$v_{\overset{-}{x}}$(%)	3.78	5.07	7.41	3.97	9.77	4.94	5.30

**Table 9:** Comparison of the thicknesses of the cured laminates (where $\overset{-}{x}$ is the arithmetic mean, and $s_{\overset{-}{x}}$ is the standard deviation of the data set).

Comparison of Thickness (mm)							
	Op0	Op1	Op2	Op3	Op4	Op5	MStd
$\overset{-}{x}$	2.34	2.09	2.08	2.16	2.27	2.23	2.31
$s_{\overset{-}{x}}$	0.08	0.04	0.03	0.04	0.06	0.06	0.05
CPT	0.260	0.232	0.231	0.240	0.252	0.248	0.256

## Data Availability

The data presented in this study are available on request from the corresponding author.
